# A Study on the Failure Behavior and Force Transmission of Composite Skin-Stringer Structures Under a Compressive Load

**DOI:** 10.3390/ma18061380

**Published:** 2025-03-20

**Authors:** Guoyang Zhao, Jian Shi, Wei Xu, Nan Sun, Jianjiang Zeng, Guang Yang, Kun Song, Jie Zheng

**Affiliations:** 1 School of Aviation Maintenance Engineering, Chengdu Aeronautic Polytechnic, Chengdu 610100, China; zhaoguoyang@cap.edu.cn (G.Z.); xuwei1395201@126.com (W.X.); 2 College of Aviation Engineering, Civil Aviation Flight University of China, Guanghan 618307, China; 3 Department of Electrical and Computer Engineering, University of Alberta, Edmonton, AB T6G 2V4, Canada; nsun6@ualberta.ca; 4 College of Aerospace Engineering, Nanjing University of Aeronautics and Astronautics, Nanjing 210016, China; 5 School of Fiber Engineering and Equipment Technology, Jiangnan University, Wuxi 214000, China; gyang@nuaa.edu.cn; 6 School of Mechanical and Power Engineering, Nanjing Tech University, Nanjing 211816, China; ksong@njtech.edu.cn; 7 Department of Mechanical Engineering, University of Alberta, Edmonton, AB T6G 2R3, Canada

**Keywords:** failure analysis, composite skin-stringer structures, load transfer, Hashin initiation criteria, Tserpes degradation law

## Abstract

Carbon fiber-reinforced composite stringers, which support aircraft skins in resisting tensile, compressive, and shear loads, are widely used in aircraft structures. These composite structures play a crucial role in enhancing the performance and safety of the structural integration of aircrafts. To better understand the load-bearing capacity of composite stringer structures, this study developed a novel model to study the complex failure and load transmission behavior of T800/3900S-2B fiber-reinforced composite skin-stringer structures under compressive loading. Compression strength tests were conducted on a composite stringer/skin structure, and a three-dimensional FEM was developed using Abaqus/Standard 2022. The model incorporated the modified 3D Hashin initiation criteria and Tserpes degradation law through a UMAT subroutine, which can effectively capture the in-plane ply failure and interlaminar damage. The results revealed a high degree of similarity between the load–displacement curves and failure modes (i.e., matrix compressive cracking, fiber compressive failure, and fiber–matrix shear-out failure) obtained from the simulations and those from the experiments. This study provides an efficient and accurate model to simulate the failure and load transfer of composite skin-stringer structures, offering significant advancements in understanding and predicting the behavior of these critical components.

## 1. Introduction

Carbon fiber-reinforced composite stringers are typically crucial longitudinal components within the wing structure of modern civil aircrafts. Functionally, they generally provide essential support to the aircraft skin, effectively preventing significant local deformation under external loads. Moreover, these stringers usually play a vital role in enhancing the shear resistance capacity and compression stability of the skin. The composite H-shaped short stringer is often used as the main load-bearing structure. When subjected to compressive loads and subsequent damage, it frequently displays intricate alterations in force transmission paths and damage behavior, as reported in references [1,2,3]. Given these complex characteristics, the in-depth study of H-shaped short stringers is significant in aircraft structural design, as emphasized in references [4,5].

Pevzner et al. [6] improved an extended effective width method that is suitable for analyzing laminated composite stringer-stiffened circular cylindrical panels. Their study developed the relevant MATLAB code to analyze the bending buckling of the stiffeners, torsional buckling, combined bending and torsion buckling, and local buckling of the stringers. Moreover, the efficiency of this engineering method in the initial design stage was verified through comparison with experiments. Christian [7] introduced some engineering analysis methods regarding the buckling and post-buckling of thin-walled composite laminated beams. Badalló et al. [8] studied the optimization of a T-shaped stringer that is commonly used in CFRP-stiffened panels by employing three common genetic algorithms. The objectives of the optimization were to minimize the mass and maximize the critical buckling load, and the most suitable algorithm for that kind of problem was obtained. Wagner and Balzani [9] utilized the finite element method to investigate delamination and skin-stringer separation phenomena in stringer-stiffened fiber-reinforced composite shells. Vescovini et al. [10] analyzed the post-buckling response and failure of multi-stringer panels using the finite element method with three levels of approximation and obtained a deeper understanding of the effect of modeling parameters such as geometric and material parameters. Shi et al. [11,12,13] employed a semi-analytical multiscale algorithm to conduct progressive damage analysis on the composite stringers and the open-hole plates extracted from the webs of the stringers. This semi-analytical multiscale algorithm significantly enhanced the efficiency of multiscale calculations while ensuring computational accuracy. The analysis results obtained were in excellent agreement with the experimental results. Bnisch et al. [14] proposed a semi-analytical analysis method for the stress fields in composite skin-stringer junctions under a bending load. Compared with the results from finite element modeling, the stress results obtained by this semi-analytical approach were more accurate, and the computational time was only a small fraction of that required by finite element methods. Some fully analytical approaches [15,16,17] have been devised to analyze the post-buckling behavior of stiffened composite panels, which can rapidly calculate the failure loads. However, their principal shortcoming is that the accuracy of the result is compromised. The progressive damage method was employed in the literature [18,19] to conduct a detailed examination of the failure mechanisms and ultimate strength characteristics of composite stringers, and the analysis results exhibited a high degree of consistency with the experimental data. Recently, an increasing number of numerical studies [20,21,22] have been conducted to investigate the skin-stringer separation in stiffened composite panels and explore the influence of intra-laminar damages on the debonding evolution.

In summary, many researchers have studied the mechanical properties of either stringers or panels formed by stringers and skins. However, there are limited studies on the crushing behavior and force transmission paths of short co-cured skin-stringer structures under compressive loading. This paper performs experimental and numerical analyses of short H-shaped composite skin-stringer structures. The progressive damage method based on a modified 3D Hashin initiation criterion and Tserpes degradation law through a UMAT subroutine is used to predict the load–displacement curve, ultimate load, failure modes, and force transmission of the fiber-reinforced composite structures under compressive loading. Then, the analysis results are compared with the test results.

## 2. Failure Criteria and Material Property Degradation Rules

Composite structures exhibit complex and diverse failure modes. Failure criteria must account for stress coupling across material directions and differentiate damage states. Based on this aim, several failure criteria with excellent performance, such as the Chang-Chang [23,24], Hashin [25], and Puck [26] failure criteria, are widely acknowledged as notable and effective criteria. The macroscopic 3D Hashin failure criteria considering delamination and fiber–matrix shear-out [27] are used in the current study to determine the initial failure within the composite laminate of the stringer, as shown in Equations (1)–(7). It is generally believed that the mechanical behavior of the fiber is linearly elastic and brittle before fiber failure occurs. Therefore, the maximum stress criterion is adopted for the initial fiber failure.

The fiber tensile failure, for *σ*_11_ > 0, can be determined as follows:

(1) $$\frac{\sigma_{11}}{X_{T}}\geq 1,$$

The fiber compressive failure, for *σ*_11_ < 0, can be determined as follows:

(2) $$\frac{\sigma_{11}}{X_{C}}\geq 1,$$

The matrix tensile cracking, for *σ*_22_ > 0, can be determined as follows:

(3) $${\left(\frac{\sigma_{22}}{Y_{T}}\right)}^{2}+{\left(\frac{\sigma_{12}}{S_{12}}\right)}^{2}+{\left(\frac{\sigma_{23}}{S_{23}}\right)}^{2}\geq 1,$$

The matrix compressive cracking, for *σ*_22_ < 0, can be determined as follows:

(4) $${\left(\frac{\sigma_{22}}{Y_{C}}\right)}^{2}+{\left(\frac{\sigma_{12}}{S_{12}}\right)}^{2}+{\left(\frac{\sigma_{23}}{S_{23}}\right)}^{2}\geq 1,$$

The delamination in tension, for *σ*_33_ > 0, can be determined as follows:

(5) $${\left(\frac{\sigma_{33}}{Z_{T}}\right)}^{2}+{\left(\frac{\sigma_{13}}{S_{13}}\right)}^{2}+{\left(\frac{\sigma_{23}}{S_{23}}\right)}^{2}\geq 1,$$

The delamination in compression, for *σ*_33_ < 0, can be determined as follows:

(6) $${\left(\frac{\sigma_{33}}{Z_{C}}\right)}^{2}+{\left(\frac{\sigma_{13}}{S_{13}}\right)}^{2}+{\left(\frac{\sigma_{23}}{S_{23}}\right)}^{2}\geq 1,$$

The fiber–matrix shear-out, for *σ*_11_ < 0, can be determined as follows:

(7) $${\left(\frac{\sigma_{11}}{X_{C}}\right)}^{2}+{\left(\frac{\sigma_{12}}{S_{12}}\right)}^{2}+{\left(\frac{\sigma_{13}}{S_{13}}\right)}^{2}\geq 1,$$

where *σ_ij_* (*i*, *j* = 1, 2, 3) represents the normal and shear stress component; *X_T_*, *Y_T_*, and *Z_T_* are the tensile strengths of the unidirectional ply in the normal direction. Similarly, *X_C_*, *Y_C_*, and *Z_C_* denote the three normal compressive strengths. Additionally, *S_ij_* (*i*, *j* = 1, 2, 3) is the shear strength of the unidirectional ply. Once the failure criteria are satisfied, the stiffnesses of the failed elements are reduced.

The sudden material property degradation rules of Tserpes [28,29] are adopted to reduce the material stiffness of the failed elements. Compared with other degradation schemes, these rules define a progressive three-dimensional stiffness degradation model based on the physical failure mechanism of the Chang-Chang model [23] and incorporate the mechanisms of delamination and fiber–matrix shear-out failure, as shown in Table 1. To avoid the singularity problem of the stiffness matrix during the calculation, 0 in the degradation criterion is set to a very small value of 10^−9^.

## 3. Uniaxial Compressive Experiment

**Figure 1:**
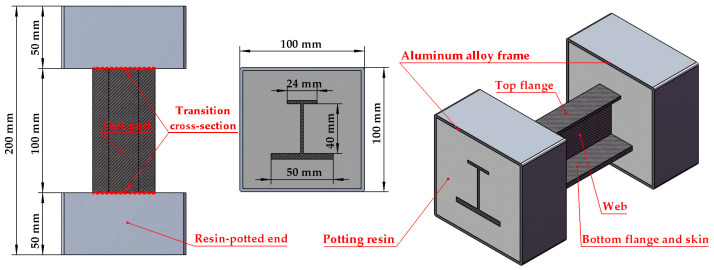
Geometric model of the test specimen, illustrating key structural components and dimensions. The specimen features a central test area with a transition cross-section, flanked by resin-potted ends for load application. A potting resin secures the structure, while an aluminum alloy frame provides external reinforcement. The internal H-section consists of a top flange, web, and bottom flange with skin.

The specimen of the stringer is composed of four parts: the top flange, web, bottom flange, and skin. The bottom flange and the skin are bonded together, and this bonded structure can be treated as an integrated laminated plate. The manufacturing processes and quality of these specimens are strictly controlled to reduce errors caused by possible defects, such as fiber discontinuities. To ensure a uniform load distribution, two resin-potted ends, each consisting of an aluminum alloy frame and a potting resin region, are attached at both ends of the specimen. The geometric dimensions of the specimen are presented in Figure 1. Specifically, the total length of the specimen measures 200 mm, which encompasses two 50 mm long reinforced sections and one 100 mm long test section. The cross-sectional widths of the top and bottom flanges are 24 mm and 50 mm, respectively, and the height of the web is 40 mm.

Table 2 describes the stacking sequences, the thicknesses of the stringer components, and the number of plies. The thickness of each stringer ply is 0.19 mm. So, the thickness of each stringer component is equal to the number of plies multiplied by the thickness of a single ply.

The laminate material of the stringer is T800S/3900-2B, which is widely used in the main bearing structures of civil aircrafts. Table 3 presents the elastic modulus and strength parameters.

**Figure 2:**
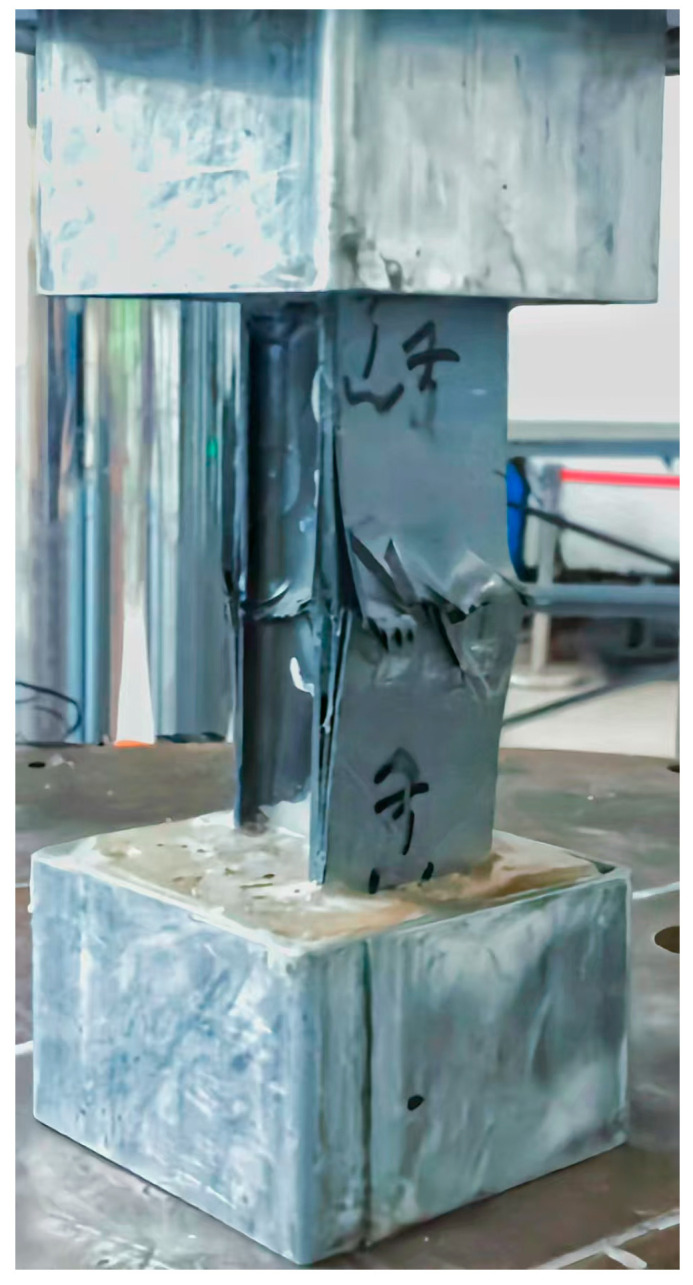
The final failure state of the stringer. The failure includes web buckling, flange delamination, and localized fractures, indicating progressive damage accumulation and ultimate load-bearing capacity loss.

The uniaxial compression tests of the H-shaped stringer specimen were carried out on an Instron hydraulic test machine at a room temperature of 25 °C, and the displacement load was applied at a rate of 2 mm/min. Figure 2 presents the final failure state of the composite stringer, which showed that the catastrophic failures included delamination, fiber, and matrix compression failures. These failures occurred at the midpoint of the specimen, indicating that the resin-potted end design in this experiment effectively prevented a concentration of stress near the loading end caused by a non-uniform load application.

**Figure 3:**
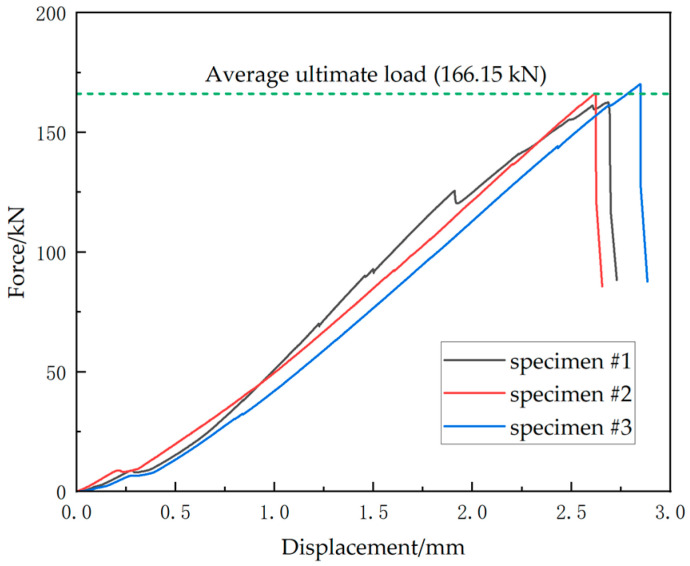
Load–displacement curves from the uniaxial compression test, showing the force–displacement response of three specimens. The applied forces and measured displacements are in the compressive direction. The average ultimate load is 166.15 kN, as marked by the dashed green line.

Figure 3 shows the load–displacement curve of the uniaxial compression tests. During initial loading, some noises occurred at the resin-potted ends, which were likely generated as some small gaps within the potting region were being compacted. This corresponds to the stage of extremely low modulus at the initial part of the load–displacement curve. The specimens’ average ultimate load is 166.15 kN, with a corresponding average displacement of 2.72 mm. Subsequently, a brittle fracture occurred in the middle of the specimen, resulting in a rapid load decline.

## 4. Finite Element Model Description

**Figure 4:**
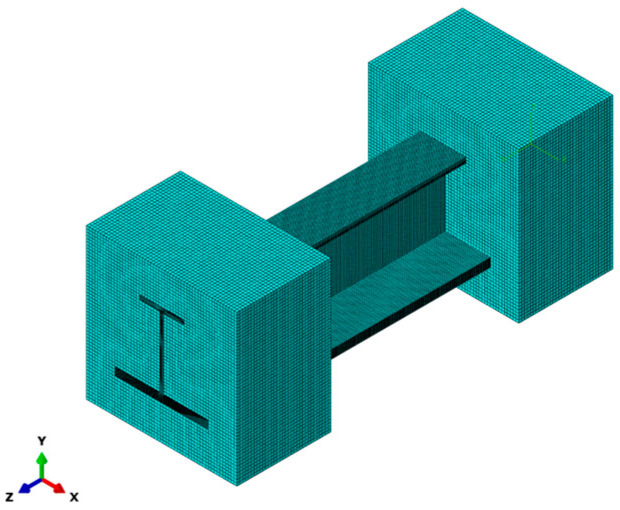
FEM mesh of stringer structure.

The element type C3D8R was selected for the FEM of the composite stringer. The element size of the two resin-potted ends was 1.5 mm, and each stringer ply was meshed separately. Specifically, the mesh sizes of each ply in the thickness direction and in-plane direction were set to 0.19 mm and 0.75 mm to balance computational efficiency and accuracy. The finite element model is presented in Figure 4. In the model, one end of the specimen was securely fixed, while a displacement-controlled load was applied to the other end. A reference point was set at the geometric center of the stringer’s loading surface to ensure a uniform distribution of the load acting on the structure. Additionally, a multi-point constraint (MPC) was employed. This MPC tied the reference point to all the nodes of the loading surface. The stringer’s axial displacement load was then applied to this reference point. This arrangement enabled the load to be applied and facilitated the monitoring and output of the load–displacement history data during the analysis process.

## 5. Results and Discussion

The three-dimensional FEM was established using Abaqus/Standard 2022, which integrated the modified 3D Hashin initiation criterion and Tserpes degradation law through a UMAT subroutine. Solution-dependent state variables (SDVs) within the Abaqus UMAT subroutine were used in the simulation. Specifically, the failure parameters are represented by these SDVs. SDV1 to SDV7 correspond to failure criterion Formulas (1)–(7). When an SDV is 0, its corresponding element is unfailed, and the value of 1 indicates that element failure has occurred.

**Figure 5:**
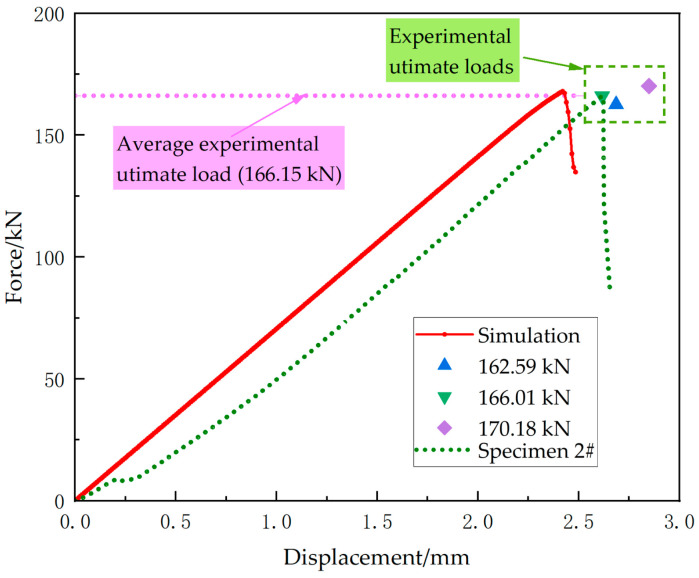
Load–displacement response from the uniaxial compression simulation. The simulated force–displacement curve (solid red line) is compared to the experimental results, including individual experimental ultimate loads (marked symbols) and the experimental load–displacement curve of specimen 2# (green dashed line). The average experimental ultimate load (166.15 kN) is indicated by the dashed purple line, demonstrating good consistency between the simulated and experimental data.

Figure 5 shows the load–displacement curve derived from the simulation. The ultimate load of this curve is 168 kN. The results reveal a 1.11% relative error between the simulated and experimental average ultimate loads. Since the simulation model does not consider actual factors in the reinforced ends, such as the voids, the load–displacement curve exhibits more distinct linearity and increased brittleness during failure. The displacement corresponding to the ultimate load in the simulated curve is 2.42 mm, slightly smaller than its experimental counterpart, and this minor deviation is thus deemed reasonable.

**Figure 6:**
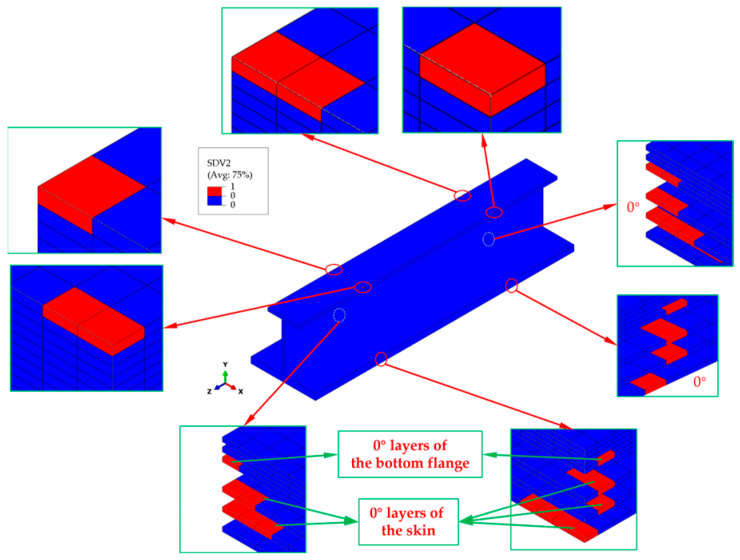
Initial fiber compression failure distribution in the stringer structure, highlighting affected 0° layers in the bottom flange and skin. The red regions indicate failed elements, showing failure initiation at the transition cross-sections. The insets provide a detailed view of localized damage, revealing stress concentration points leading to progressive failure.

When the external load reaches 149 kN, initial compressive fiber damage occurs on the outer edges of the top and bottom flanges at the transition cross-sections of the stringer. The failure modes mainly include fiber compression failure in the 0° ply, matrix compression failure in the ±45° plies, and fiber–matrix shear-out failure in the 0° ply. The potting regions constrain all degrees of freedom (DOFs) of the two clamped parts of the stringer, except the longitudinal DOF. This constraint effectively avoids abnormal stress concentration at the contact regions between the stringer ends and the fixture block of the test machine. The maximum stress values occur at the transition cross-sections of the stringer. Among them, the longitudinal normal stress *σ*_11_ in the 0° ply is significantly larger than that in other plies. This indicates that the 0° plies mainly bear the compressive load. Therefore, initial fiber compression failure occurs first in the 0° plies, and the failure locations are at the transition cross-sections, as shown in Figure 6. Only a small number of elements in the top flange have failed, while more elements have failed in the 0° ply of the bottom flange and all 0° plies of the skin, and no fiber compression failure is observed in the web. The percentage of failed elements in the upper flange is nearly 20%, whereas failed elements of the bottom flange and skin contribute to approximately 80% of the total structural failure. The above-mentioned failure states indicate that the 0° plies of the bottom flange and the skin mainly bear the compressive load. Moreover, the bottom flange and the skin achieve the maximum thickness and the strongest load-bearing capacity, which proves that the force transmission path before the occurrence of initial damage is reasonable.

**Figure 7:**
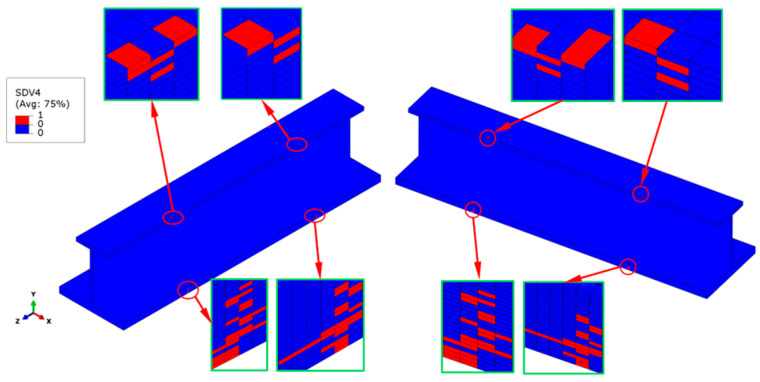
Matrix compressive failure distribution at a 149 kN load. The red regions indicate failed elements, located in the top flange, bottom flange, and skin near the transition areas. The insets provide a closer view of localized failures, showing progressive damage that is spread under increasing compressive loads.

In the same simulation case, when the external load reaches 149 kN, some matrix compression failures are generated on both ends’ cross-sections of the stringer test part, as shown in Figure 7. The initial matrix compression failure occurs earlier than the fiber compression failure, and there is a certain degree of damage propagation. The matrix compression damage mainly occurs in the non-0° plies of the skin and the bottom flange. The main reason is that these plies are subjected to a relatively large in-plane transverse compressive stress, *σ*_22_, which is caused by the compressive load. A few elements have triggered matrix compression failures on the top flange, while no matrix failure is observed on the web. This situation also indicates that the bottom flange and the skin are the main parts bearing the compressive load.

**Figure 8:**
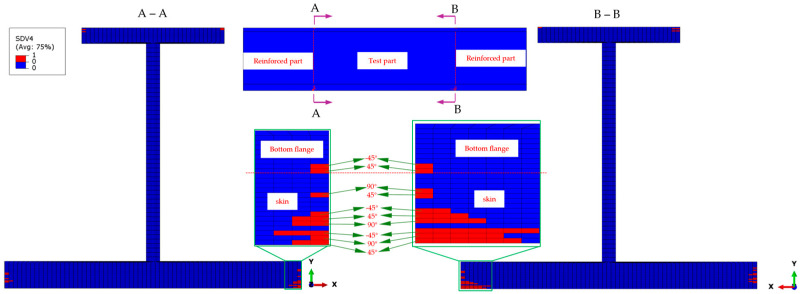
Matrix compressive failure distribution at the transition cross-section under a 149 kN load, highlighting damage progression in the bottom flange and skin. The red regions indicate failed elements, showing failure initiation at the outer edges and gradual propagation toward the web due to increased compressive stress. The insets provide a detailed view of localized damage.

In addition to spreading axially along the stringer, the matrix compression damage in the non-0° plies of the skin and the bottom flange also propagates transversely, as shown in Figure 8. The matrix failure initiates in the elements at the outer edges of the transition cross-sections, as shown in the section views A-A and B-B. The top and bottom flanges deform in thickness and the transverse directions under compression, with the web connecting them leading to a higher in-plane shear stress field. As the compressive load increases, the damage at the outer edges of the flange transition cross-sections gradually propagates towards the web.

**Figure 9:**
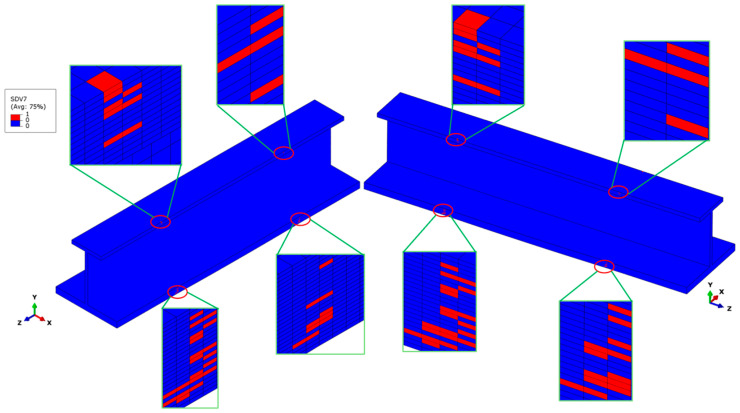
Fiber–matrix shear-out failure distribution at a 149 kN load, highlighting the failure concentration along the edges of the transition cross-sections. The red regions indicate failed elements. The insets provide a detailed view of the localized shear failure propagation, illustrating its role in structural degradation.

The other failure modes mainly include the fiber–matrix shear-out failure in the initial failure stage, as shown in Figure 9. The shear failure elements are localized on the edges of the transition cross-sections of the stringer test part. The 0° ply of the top and bottom flanges experiences significant stress, *σ*_11_, causing the stress components to satisfy the shear failure criteria and trigger fiber–matrix shear-out failure.

**Figure 10:**
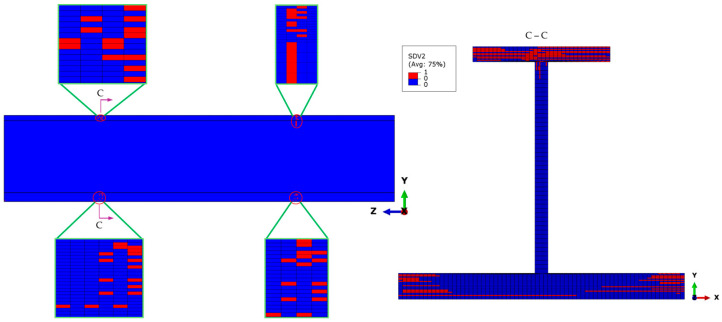
The fiber compressive failure distribution under the ultimate load of 168 kN, highlighting significant failure expansion in the transition cross-sections. The red regions indicate failed elements, primarily concentrated in the top flange, bottom flange, and skin, while the web experiences minimal failure. The insets provide a detailed view of the localized damage.

Under the ultimate load of 168 kN, the fiber compressive failure propagates extensively within the transition cross-sections at both ends of the stringer test part, as shown in the section views C-C of Figure 10. Fiber compressive failure occurs in most of the plies of the top flange cross-section, which causes the specimen to reach peak load. Relatively large areas with fiber failure also exist in the bottom flange and the skin, but the extent of the lateral damage is minimal, so they still retain a substantial load-bearing capacity. In the web of the stringer, however, there are only isolated regions with fiber compressive failure in the 0° plies. Therefore, the subsequent external load is mainly borne by the web, the bottom flange, and the skin.

**Figure 11:**
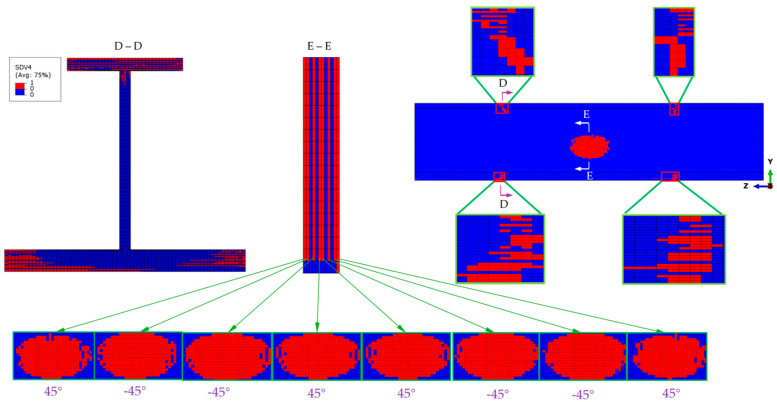
Matrix compressive failure distribution under the ultimate load, primarily affecting the ±45° plies. An elliptical failure zone forms in the middle of the web, as shown in the sectional views. The insets provide a detailed view of the localized damage, highlighting the failure propagation across the transition cross-sections.

At the ultimate load, the matrix compressive failure that mainly occurs in the ±45° plies also propagates extensively, with the most severe failure being observed at the top flange, as shown in the section view D-D of Figure 11. Extensive damage is also observed on both sides of the transition cross-sections of the stringer test part, but most of the plies have not been penetrated through the thickness. Owing to the extensive fiber compressive failure in the top flange, the load-bearing capacity of the top flange is significantly reduced, thus transferring the load to the web. Therefore, an elliptical area of matrix failure appears in the middle of the web, and the failure still mainly occurs in the ±45° plies, as shown in the section views E-E.

**Figure 12:**
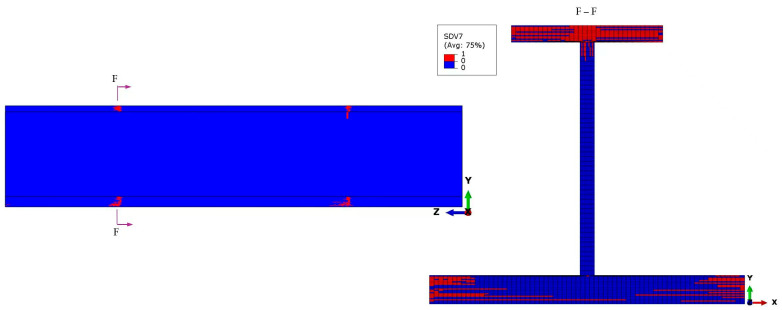
Fiber–matrix shear-out failure distribution under the ultimate load, highlighting the failure concentration at the transition cross-section, particularly in the top flange and bottom flange. The red regions indicate failed elements. Section F-F provides a detailed view, showing that while the failure has significantly progressed, it has not yet fully penetrated transversely.

Under the ultimate load, the fiber–matrix shear-out failure has propagated extensively throughout the transition cross-section of the stringer test part, as shown in the section views F-F of Figure 12. The most severe failure regions are located at the top flange, where the failure of most plies has penetrated the cross-section and extended to the upper end of the web. The fiber–matrix shear-out failure is comparable to the fiber compressive failure on the skin and the bottom flange in terms of the influence of the stress, *σ*_11_, in the direction of the fiber to these failures. However, the fiber–matrix shear-out failure in this area has not penetrated the thickness yet.

**Figure 13:**
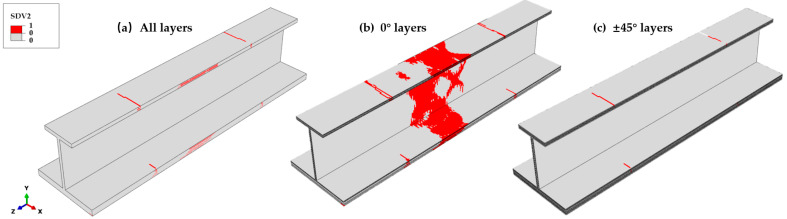
The final fiber compressive failure distribution across different ply orientations. (**a**) The failure distribution in all layers. (**b**) The 0° layers: extensive failure in the transition and central regions, where the compressive loads are primarily carried. (**c**) The ±45° layers: limited failure, mainly at transition points. The results indicate that 0° plies bear the majority of the compressive stress, leading to significant structural degradation.

After reaching the ultimate load, fiber compression failures, matrix compression failures, and fiber–matrix shear-out failures initiate at the root cross-section of the top flange test part, reducing the load-carrying capacity. Subsequently, the load-bearing function transfers rapidly to the web, bottom flange, and skin, causing damage in the FEM midsection to propagate rapidly and penetrate the entire model, forming the final failure state. Figure 13 shows the fiber compressive failure in the final state. Fiber compressive failures of the non-0° plies occur at the top and bottom root cross-sections of the stringer test part. A significant concentration of fiber compressive failures exists in the 0° ply at the middle of the specimen, as these layers primarily bear the compressive load. The location and form of the final fiber compressive failure are consistent with the results of the experiment.

**Figure 14:**
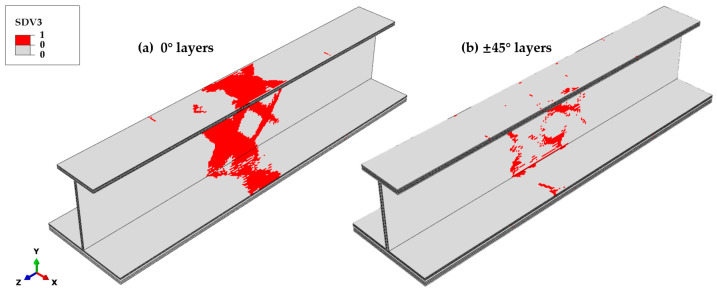
Final matrix tensile failure distribution across different ply orientations. (**a**) The 0° layers: extensive failure concentrated in the middle section. (**b**) The ±45° layers: failures are irregularly distributed across the web.

Matrix tensile failures mainly occur in the 0° ply at the middle position of the stringer, as shown in Figure 14, reducing the elastic modulus, *E*_11_, of the failed elements and causing a significant increase in strain in the fiber direction. Poisson’s ratio effects elevate the transverse strain and stress in the failed elements, leading to extensive matrix tensile failures in the 0° ply at the specimen’s midsection. The distribution of matrix tensile failures is in close agreement with that of fiber compressive failures. For the ±45° plies, matrix tensile failures are irregularly distributed on the web, with primary contributions arising from the shear stress components *σ*_12_ and *σ*_23_.

**Figure 15:**
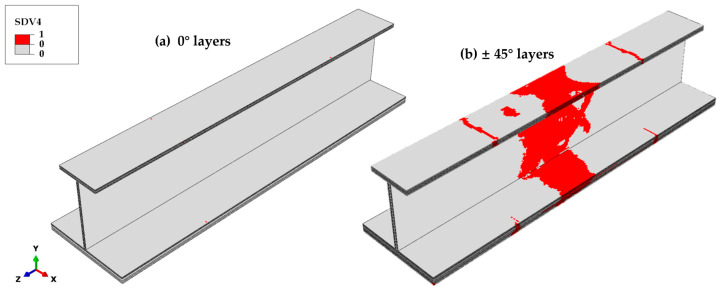
Final matrix compressive failure distribution across different ply orientations. (**a**) The 0° layers: minimal failure observed. (**b**) The ±45° layers: extensive failure concentrated in the transition cross-section and central region, while the damage propagates along the height of the web and transversely through the top and bottom flanges and skin in the central region of the structure.

Matrix compressive failure rarely occurs in the 0° ply, yet it occurs extensively in the ±45° plies, as shown in Figure 15. In the ±45° plies, the matrix compressive failure initiates at the transition cross-section of the stringer test part. Subsequently, as the load approaches the ultimate load, isolated failure regions emerge in the web midsection. Shortly afterwards, these regions expand rapidly and penetrate the web thickness. Simultaneously, transverse penetration predominantly occurs in the ±45° plies of the top and bottom flanges, as well as the central region of the skin.

**Figure 16:**
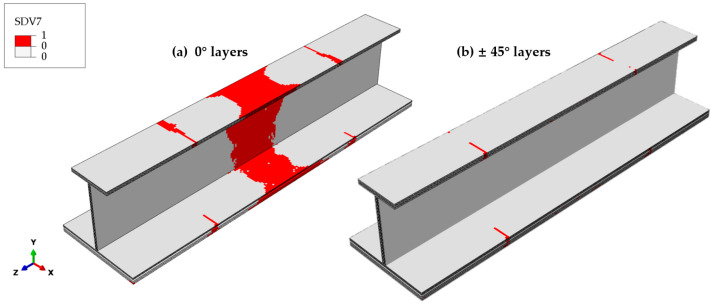
Final fiber–matrix shear-out failure distribution across different ply orientations. (**a**) The 0° layers: extensive failure in the transition and central regions. (**b**) The ±45° layers: failure is localized to the transition cross-section, without propagation to other regions.

The fiber–matrix shear-out failure is closely correlated with the stress along the fiber direction. As illustrated in Figure 16, the distribution pattern of the fiber–matrix shear-out failure in the 0° plies resembles that of the fiber compressive failure. While the longitudinal stress *σ*_11_ plays a role, the shear stresses *σ*_12_ and *σ*_23_ also drive the initiation of fiber–matrix shear-out failure. In the case of the ±45° plies, the fiber–matrix shear-out failure is localized at the transition cross-section of the test part and does not occur elsewhere. The analysis results indicate that delamination in tension and compression rarely occurs in the thickness direction due to the negligible normal stress *σ*_33_. However, fiber–matrix shear-out failures predominate in the 0° plies, while the ±45° plies exhibit minimal damage. This indicates that the shear stress difference between the 0° and ±45° plies is the primary mechanism for shear delamination.

## 6. Conclusions

Through carefully designed tests on the fiber-reinforced composite stringer and skin structure, the ultimate load, load–displacement curve, and failure modes were accurately determined. The load–displacement curve mainly exhibited brittle behavior, which is a crucial indicator of the material’s mechanical response under compressive loading. The failure modes primarily included fiber compressive failure, matrix compressive failure, and fiber–matrix shear-out failure localized at the specimen’s midsection, reflecting the complex interaction between the fiber and matrix components.

In addition to the experimental investigations, an in-depth simulation analysis was conducted to explore the damage evolution process further. Specifically, this analysis focused on three aspects: initial damage, damage under the ultimate load, and final failure state. Remarkably, the failure modes obtained from the simulation were in excellent agreement with the experimental results, validating the reliability of the simulation model. Specifically, the initial damage identified through simulation initiated at the transition cross-sections of the top and bottom flanges of the test section. As the external load gradually increased, extensive fiber and matrix compressive failures occurred at the transition cross-sections of the top flange. This accumulation of damage ultimately led to reaching the ultimate load. Consequently, isolated matrix compressive failure was observed at the web midsection. Finally, the damage propagated rapidly through the model midsection, closely matching the experimental final failure state.

The simulation process effectively revealed the intricate load transfer path during the damage progression. Due to the reinforcing effect of the potting-reinforced ends, the damage at the top flange transition cross-section of the test section was the most severe. After reaching the ultimate load, the load-bearing function was transferred to the specimen’s midsection.

This study provides a solid foundation for improving potting-reinforced end designs and optimizing specimen configuration. For instance, a more sophisticated potting-reinforced end could be designed to effectively alleviate the concentration of stress. Moreover, the ply thickness or transverse dimension of the top flange could be appropriately adjusted to enhance its load-bearing capacity, thereby optimizing the overall performance of the composite structure.

## Figures and Tables

**Table 1:** Sudden material property degradation rules.

Failure Mode	Sudden Degradation Rule
Fiber tensile failure	$E_{11}^{d}=0.07*E_{11}$
Fiber compressive failure	$E_{11}^{d}=0.14*E_{11}$
Matrix tensile cracking	$E_{22}^{d}=0.2*E_{22},E_{12}^{d}=0.2*E_{12},E_{23}^{d}=0.2*E_{23}$
Matrix compressive cracking	$E_{22}^{d}=0.4*E_{22},E_{12}^{d}=0.4*E_{12},E_{23}^{d}=0.4*E_{23}$
Fiber–matrix shear-out	$E_{12}^{d}=0$
Delamination in tension and compression	$E_{33}=E_{23}=E_{13}=0$

**Table 2:** The section sizes and ply angles of the stringer.

Section	Ply Angles/°	Number of Plies	Thickness
Top flange	45/−45/0/−45/0/45/0/0/45/0/−45/0/−45/45	14	2.66 mm
Web	45/−45/0/−45/0/45/45/0/−45/0/−45/45	12	2.28 mm
Bottom flange	45/−45/0/45/0/−45/0/−45/45	9	1.71 mm
Skin	45/90/−45/0/90/45/−45/0/−45/45/90/0/−45/90/45	15	2.85 mm

**Table 3:** Mechanical properties of T800S/3900-2B lamina, provided by Commercial Aircraft Corporation of China, Ltd., Shanghai, China.

Elastic Property	Value	Strength Property	Value
*E*_11_	154 GPa	*X_T_*	2690 MPa
*E*_22_	9 GPa	*X_C_*	1380 MPa
*E*_33_	9 GPa	*Y_T_*	93 MPa
*G*_1__2_	7 GPa	*Y_C_*	211 MPa
*G*_23_	7 GPa	*Z_T_*	93 MPa
*G*_13_	7 GPa	*Z_C_*	211 MPa
*v*_12_, *v*_23_, *v*_13_	0.3	S_12_, S_23_, S_13_	109 MPa

## Data Availability

The original contributions presented in this study are included in the article. Further inquiries can be directed to the corresponding authors.
